# Application-Specific Oxide-Based and Metal–Dielectric Thin-Film Materials Prepared by Radio Frequency Magnetron Sputtering

**DOI:** 10.3390/ma12203448

**Published:** 2019-10-21

**Authors:** Mohammad Nur-E-Alam, Wade Lonsdale, Mikhail Vasiliev, Kamal Alameh

**Affiliations:** Electron Science Research Institute, School of Science, Edith Cowan University, 270 Joondalup Drive, Joondalup, WA 6027, Australia; w.lonsdale@ecu.edu.au (W.L.); m.vasiliev@ecu.edu.au (M.V.); k.alameh@ecu.edu.au (K.A.)

**Keywords:** thin films, coatings, magneto-optics, sensors, glass, heat regulation

## Abstract

We report on the development of several different thin-film functional material systems prepared by radio frequency (RF) magnetron sputtering at Edith Cowan University nanofabrication labs. While focusing on the RF sputtering process optimizations for new or the previously underexplored material compositions and multilayer structures, we disclose several unforeseen material properties and behaviours. Among these are an unconventional magnetic hysteresis loop with an intermediate saturation state observed in garnet trilayers, and an ultrasensitive magnetic switching behaviour in garnet-oxide composites (GOC). We also report on the unusually high thermal exposure stability observed in some nanoengineered metal–dielectric multilayers. We communicate research results related to the design, prototyping, and practical fabrication of high-performance magneto-optic (MO) materials, oxide-based sensor components, and heat regulation coatings for advanced construction and solar windows.

## 1. Introduction

Among all the physical vapor deposition techniques, magnetron sputter deposition is one of the most common technologies used to grow thin-film materials and coatings for various existing and modern nanotechnological applications. Sputter deposition of thin films has a long track record of development history in vacuum technology since the 1800s [1,2,3,4,5,6,7,8,9,10]. Many dedicated research groups and scholars were involved in the development of the modern phase of magnetron sputtering technology, although the journey of the magnetron sputtering process is still considered far from its ultimate development [6,8]. However, nowadays, magnetron sputtering is considered one of the most effective processes for the deposition of a wide range of high-quality thin-film materials either in single or multilayer form, including metals, dielectrics, metal oxides of complex stoichiometry, and nanoparticles. A vast number of sputtered material types were shown to be suitable for various existing and emerging nanotechnology-related applications, and attractive for many rapidly growing diverse market sectors. Sputtering processes allow thin-film materials to be deposited either on bare substrates or on top of any structured systems, e.g., photonic or magneto-photonic crystals, usually without having any unpredictable thickness uniformity or stoichiometry issues with the grown films. Thin-film materials, especially radio frequency (RF) magnetron-sputtered thin films, were originally considered useful mainly in electronics and semiconductor devices; however, sputtered thin films have also been found to be applicable within a wide range of fields, such as optoelectronics, energy, mechanical/chemical, optical coatings, life sciences, and others [9,10,11,12,13,14,15,16,17,18,19,20,21,22,23,24,25,26,27,28,29,30]. In this nanotechnological era, the sputter deposition of thin films becomes a rather ubiquitous and actively growing field of human endeavour, integrating the fundamental and important scientific areas of research, process development, deposition system design, and new product manufacturing. This trend is expected to continue into the periods extending far into the foreseeable future. The reasons behind the rapid growth of the sputtering industry over other thin-film manufacture techniques include (i) the possibility of using large-area sputtering targets that provide uniform film thickness over large-area substrates; (ii) flexibility of growing thin- or extremely ultrathin-film materials of various composition types (even complex stoichiometry types, e.g., oxide mix-based iron garnet precursors) on different substrate types; (iii) ease of controlling the film thickness by calibrating the deposition run times and other parameters; (iv) better layer-to-substrate adhesion quality, compared to techniques such as thermal evaporation. Furthermore, the successful establishment of deposition technologies for thin polymer films and metal/plasma polymer nanoparticles fabrication using polymer or metal–polymer composite targets in RF magnetron sputtering systems by optimizing the process gas chemistries (e.g., argon, nitrogen), shows the diversity of thin-film types grown by RF magnetron sputtering [31,32,33,34]. 

**Figure 1:**
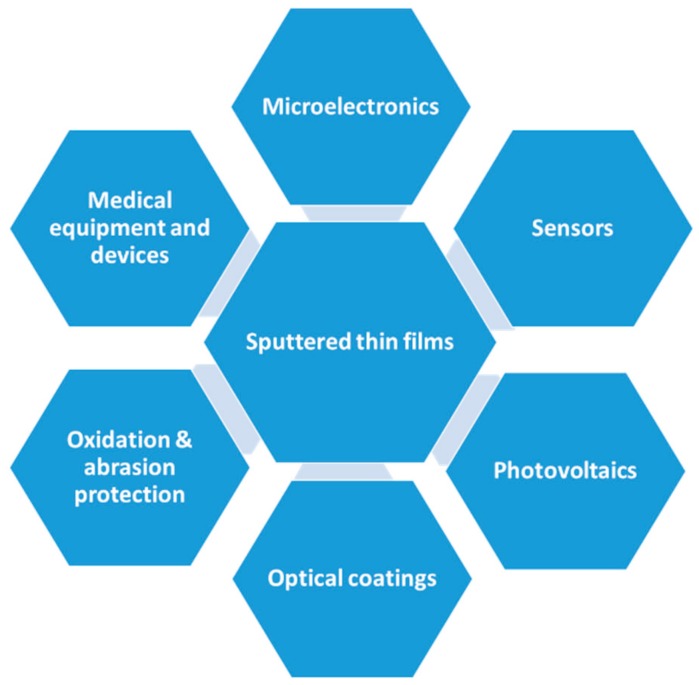
Spectrum of modern nanotechnological application areas reliant on sputtered thin-film materials.

Another important area of ongoing research is the development of sputtered metallic and the high-entropy alloy-type (either pure metal-based or metal-composite alloy, or metallo-ceramic) thin-film materials for various emerging applications, such as radiation shielding and magnetostatic shielding, reported in [35,36,37]. Figure 1 presents a wide range of possible application areas of sputtered thin films for continued exploration by future generations. 

Based on the current and possible future emerging applications, the global market of sputtered thin films can be subdivided into many segments. According to the report [38] made by PR Newswire in 2017, the sputtered thin films market volume, including sputtering-related equipment, raw materials, and services, amounts to approximately one-quarter of the total estimated (over US $70 billion) value of global markets of thin-film technologies in 2016, and is expected to grow at a significant rate. However, according to the MarketWatch report published in 2018, the thin-film materials market is expected to witness a substantial growth from 2018 to 2024, due to the demand for miniaturization of thin-film-related technologies, devices, and products. In addition, the increasing number of applications and end-use industry practices are also expected to propel the growth of the thin-films product markets in the near future [39].

In this paper, we discuss the experimental results selected from a decade-long lab-based work, during which the RF sputtering synthesis of different thin-film material types and multilayer structures have been explored extensively. We also present and discuss the specific material development results and functional material properties of interest for several specific applications. The organization of the article is as follows. Section 2 introduces the background study of Bi-substituted iron garnet thin-film materials and multilayers together with some very recently obtained optical properties for highly bismuth-substituted iron garnets of composition type Bi_2_Dy_1_Fe_4_Ga_1_O_12_ (BiDyIG). These garnet composition types (with bismuth substitution levels above about 1.8) can only be prepared using physical vapour deposition techniques (e.g., sputtering) and have not been reported to be synthesized by crystal growth techniques such as liquid-phase epitaxy. Section 3 describes the design, fabrication, and characterization of ruthenium oxide (RuO_2_) thin films grown on rigid and flexible substrates, for sensor applications. Section 4 describes the fabrication techniques and process parameters used for growing metal–dielectric nanocomposite (MDC) layers and MDC layers contained within multilayer structures for use in next-generation, environmentally friendly, highly durable optical coatings for solar and thermal radiation control. The optical properties of these MDC thin films and multilayer building blocks containing nanocomposite-type materials are presented in this section. 

## 2. Garnets, Garnet-Oxide Composites, and Multilayer Structures Deposited by Sputtering

Natural garnets are popular as gemstones, but when they are engineered either by modifying their stoichiometry or by adjusting the fabrication process, they can possess extraordinary multifunctional properties that make them attractive for various applications, e.g., integrated photonic circuits and devices. Through nanoengineering, garnet thin films can be controlled at the atomic level to attain naturally unachievable photonic and magnetic properties that open the way towards the development of magnetophotonic and magneto-plasmonic crystals (MPCs) for a wide range of applications, including telecommunication, biomedicine, digital forensics, sensing, and data processing. Thin-film garnets have a long history that started since they were developed in 1960 for the first time for use in bubble memory device applications [39,40,41,42,43,44,45,46,47,48,49,50,51,52,53,54,55,56,57,58,59]. The properties of thin garnet films are always dependent on their fabrication methods, process parameters, and especially on the stoichiometry of the material. By varying the materials’ compositional constituents together with their optimized fabrication process parameters in RF magnetron sputtering systems, the magnetic and magneto-optic (MO) properties of garnet thin films can be tailored for specific applications. Multiple research groups worldwide [60,61,62,63,64,65,66,67,68,69] have conducted a significant number of research works using only RF magnetron sputtering to synthesize, develop, and improve many garnet thin-film material systems. Since the last decade, a number of highly bismuth (Bi) substituted iron garnet thin-film material systems, including garnet-oxide and garnet–garnet nanocomposite derivatives, have been synthesized, their sputtering process parameters optimized, and many studies of the optical, magnetic, and magneto-optical properties were reported by our group at Edith Cowan University (ECU) [70,71,72,73,74,75,76,77]. We have reported obtaining nanocrystalline garnet thin-film materials with high Faraday rotation across the visible spectral range, a record-high magneto-optic quality, very low coercive force with perpendicular magnetization, and almost in-plane magnetization types. In addition, rather unconventional magnetic switching properties have recently been demonstrated in all-garnet multilayer thin-film structures. In this section, we present the recently obtained results on the optical constants of RF-sputtered highly Bi-substituted iron-garnet precursors of nominal composition type Bi_2_Dy_1_Fe_4_Ga_1_O_12_ (BiDyIG) and garnet-oxide composites (GOC), together with a glimpse of the previously best-achieved MO properties in thin garnet films of similar compositions. The unconventional hysteresis loop shapes observed in GOC-based all-garnet multilayer structures are also presented.

### 2.1. Deposition Process Parameters for Growing Bi-Substituted Iron Garnets, GOC Precursor Layers, and GOC-Based Multilayer Structures

Several batches of garnet-oxide composite (having 17–23.3 vol. % of excess Bi_2_O_3_ content) amorphous-phase films were prepared by RF magnetron sputtering on Corning Eagle XG glass substrates. In addition, several batches of GOC-based all-garnet multilayer structures employing two different garnet-type materials were deposited onto gadolinium gallium garnet (GGG) and onto glass (Corning Eagle XG) substrates using RF magnetron sputtering. The multilayer structures were deposited in a single deposition run by sequential sputtering of layers using oxide-mix-based ceramic sputtering targets and low-pressure pure-argon plasma. The process parameters and conditions used to deposit the garnets, garnet-oxide (single) layers, and the multilayer structures are summarized in Table 1.

Note that all garnet layers and garnet–garnet multilayer structures were in amorphous phase just after deposition and were subjected to annealing crystallization using a high-temperature oven-annealing process in ambient air atmosphere optimized for each material. The optimization criterion was the maximized specific Faraday rotation in films of high surface quality. 

A common (and well-known) effect observed typically during the sputtering of most oxides and oxide-based ceramic materials (e.g., garnets, especially for pure-Ar processes) is oxygen loss, resulting in significant stoichiometry variations (mainly the oxygen content difference) between the target materials and deposited film layers. During the deposition of both the amorphous-phase garnet films and garnet-oxide composites, this manifests in the increased optical absorption in the as-deposited films, compared to the expected material data. This oxygen loss, in the case of most oxide materials and also garnets, is compensated by the diffusing oxygen during the high-temperature air-atmosphere oven annealing processes. The oxygen loss compensation can be confirmed by the reduced optical absorption in annealed oxide layers and also by the optical properties of annealed garnets closely matching these reported for garnets synthesized by other techniques (e.g., liquid-phase epitaxy or pulsed-laser deposition). 

### 2.2. Optical Properties and MO Performance of Bi-Substituted Iron Garnets, GOC Single Layers, and MO Performance of GOC-Based Multilayer Structures

**Figure 2:**
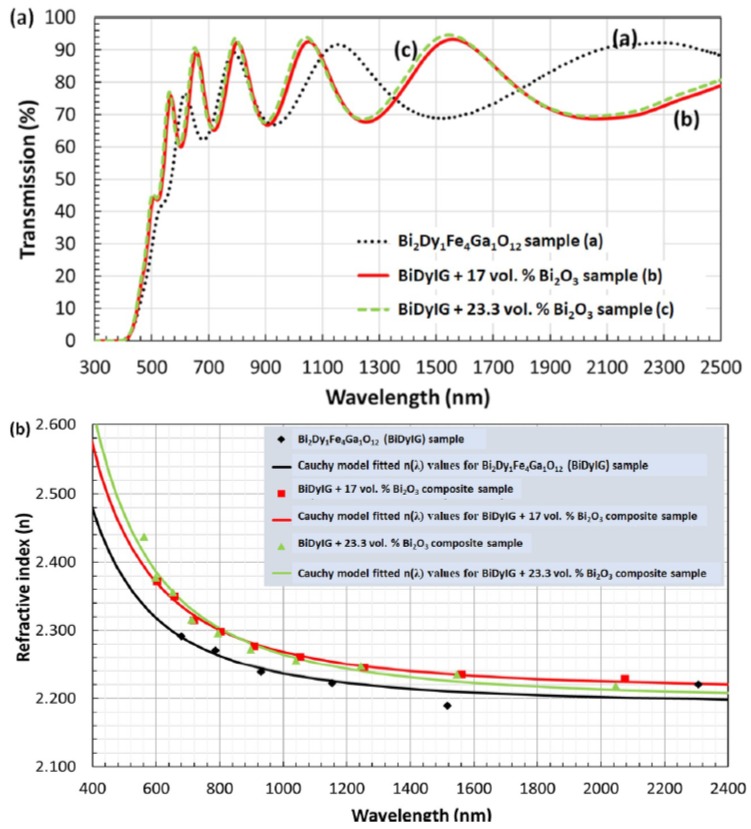
(**a**) Measured transmission spectra of as-deposited Bi_2_Dy_1_Fe_4_Ga_1_O_12_ (BiDyIG) and BiDyIG: Bi_2_O_3_ (17 and 23.3 vol. %) composite thin films, (**b**) derived refractive index dispersion spectra for Bi_2_Dy_1_Fe_4_Ga_1_O_12_ (BiDyIG) and BiDyIG:Bi_2_O_3_ (17 and 23.3 vol. %) composite thin films where the solid curves were determined according to Cauchy dispersion relationship.

Figure 2a shows the measured transmission spectra of as-deposited Bi_2_Dy_1_Fe_4_Ga_1_O_12_ (BiDyIG) garnet and BiDyIG:Bi_2_O_3_ (17 and 23.3 vol. %) composite thin-film layers prepared on glass substrates by using an RF magnetron cosputtering process. These particular garnet types possessed MO garnet compositions (and optical properties) of special importance in common application areas, e.g., Faraday rotators, ferromagnetic resonance-based microwave devices, MPCs, and MO imagers. In the cosputtering process, two separate oxide-mix-based ceramic targets (Bi_2_Dy_1_Fe_4_Ga_1_O_12_ and Bi_2_O_3_) were used and the materials volumetric fraction ratios were controlled and calculated from their partial deposition rates. The details of several GOC layer deposition technologies and sputtering process parameters are described in [70,72,73,74]. From the thickness-dependent transmission-wave interference fringe intensities (measured over a wide range of wavelengths in all samples, Figure 2a), using a new combinatorial approach, the optical constants of amorphous garnet precursor layers are calculated and refined, together with the film thickness confirmation [77]. This combinatorial approach employs a custom-built spectrum-fitting software in conjunction with the Swanepoel envelope method (SWEM). The following operational steps are conducted for the evaluation of optical constants: (i) Calculation of the refractive index data and film thickness using the SWEM method [78]; (ii) use of the calculated index data (from SWEM), in conjunction with least-square fitting to derive the real part of the index from Cauchy’s formula (n (λ) = a + b/λ^2^); (iii) use of the refractive index data (derived from Cauchy’s formula) in magnetic photonic crystal (MPC) analysis software [79] to model and fit the measured transmission spectra; and (iv) from this fitting process, reconfirm the film thicknesses and derive the absorption coefficient spectra of the films. The wavelength-dependent index (n) data (calculated based on Cauchy’s dispersion formula) for the amorphous garnet and garnet-oxide precursor thin layers deposited on glass (Corning Eagle XG, 0.5 mm thick) substrates yield the following spectral dependency for BiDyIG sample, n = 2.1906 + 0.0459/λ^2^, and for BiDyIG:Bi_2_O_3_ (17 vol. %) composite sample, n = 2.2105 + 0.0576/λ^2^. For BiDyIG:Bi_2_O_3_ (23.3 vol. %) composite sample, the index dispersion function n = 2.1961 + 0.0683/λ^2^ was obtained.

The calculated refractive indices at different wavelengths derived from Cauchy’s relation as well as the calculated refractive index data points using the SWEM method were plotted and compared (as shown in Figure 2b). It can be noted that the refractive index (n) is in the range of 2.2–2.6 in the visible region and decreases with increasing wavelength. In the long-wavelength region (above 1600 nm), the refractive index seems to be asymptotically approaching a constant value. The volumetric fraction of excess Bi_2_O_3_ introduced into the BiDyIG system helps increase the refractive index of the composite films; however, this also shifts the absorption edge towards the shorter wavelength region (as seen in Figure 2a). Note that there are always significant differences that exist between the optical constants of the crystallized garnets and oxide-mix-based amorphous precursor layers. Therefore, it is also important to check the conformity of layer(s) to their designed spectral transmission specifications before annealing, especially when developing functional multilayer structures, such as MPC.

**Figure 3:**
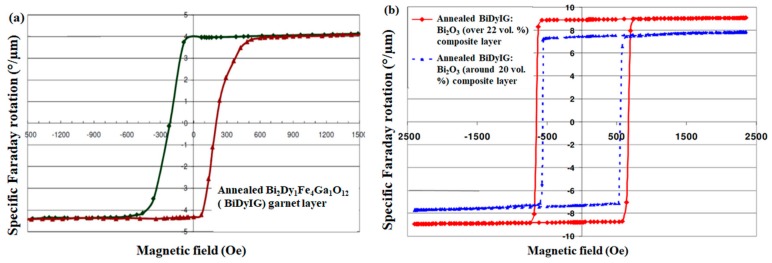
Hysteresis loops of specific Faraday rotation at 532 nm measured in sputtered garnet films. (**a**) Annealed (nanocrystalline) Bi_2_Dy_1_Fe_4_Ga_1_O_12_ (BiDyIG) sample, and (**b**) annealed BiDyIG: Bi_2_O_3_ composite-type oxide-diluted thin films [66].

In our previous work, we observed the effects of excess Bi_2_O_3_ addition to core materials within each garnet-related oxide mix (a generic composition type can be defined using a formula description such as (Bi, Y, Dy, Lu, …)_3_(Fe,Al,Ga,…)_5_O_12_) on their annealing crystallization behaviour and the final optical/MO properties. It was reported that an optimized amount of extra Bi_2_O_3_ content addition could significantly improve the specific Faraday rotation, optical transparency, and optical absorption coefficient across the visible spectral range, thus leading to achieving record-high MO quality factors in suitably annealed garnet-oxide composite thin films [70,72]. Figure 3 shows the typical magnetic hysteresis loop properties of annealed garnet and GOC films measured through specific Faraday rotation at 532 nm using a Thorlabs PAX polarimeter system in conjunction with a custom-made calibrated electromagnet. Notably, the annealed BiDyIG sample showed a nearly square-shaped hysteresis loops of Faraday rotation (Figure 3a), whilst the annealed GOC films often showed practically perfectly “square” shapes of hysteresis loop (Figure 3b) with perpendicular magnetic anisotropy, high (close to 100%) remnant magnetization, and very sensitive magnetic switching behaviour.

The strong uniaxial magnetic anisotropy in BiDyIG:Bi_2_O_3_ nanocomposite films, together with some degree of control over the coercive force and switching field properties (as shown in Figure 3b) and excellent optical and magnetic properties, make them very attractive and promising for a large range of optoelectronic, photonics-related, sensing, and MO imaging applications. A particularly notable result was the ultrahigh magnetic-field sensitivity observed in the magnetic switching behaviour of nanocomposite-based high-performance MO materials.

**Figure 4:**
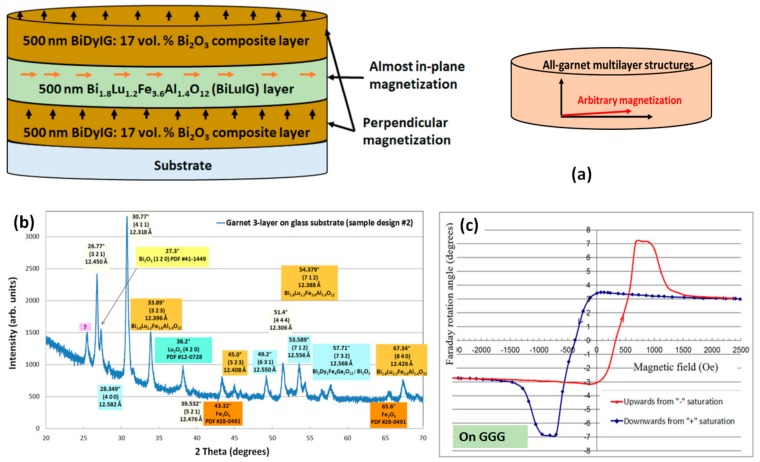
The schematic diagram of all-garnet multilayer structures together with the predicted layer-specific magnetization vector directions (**a**); XRD analysis results obtained from a multilayer garnet structure prepared on a glass substrate (**b**); the unconventional magnetic hysteresis loop of Faraday rotation (°) measured using green laser light source (**c**).

We observed somewhat unconventional and remarkable magnetic switching properties for the first time in RF magnetron-sputtered all-garnet multilayer thin-film structures, in which magneto-soft layers were sandwiched in-between two magneto-hard garnet material layers of identical composition type [80]. Both garnet material types possessed very large specific Faraday rotation across the visible spectral range (≈6 °/µm at 532 nm and ~1.6 °/µm at 635 nm [70,72]) and high magneto-optic (MO) quality. The magnetic switching behavior of trilayers (illustrated in Figure 4a) was characterized by way of measuring their Faraday rotation hysteresis loops. The samples were prepared on glass (Corning Eagle XG) as well as GGG (111) substrates, and the sputtering deposition was followed by postdeposition annealing crystallization (heat treatment process, 1 h at 570 °C) conducted in air atmosphere. The amount of extra bismuth oxide content cosputtered from a separate target (17 vol. %) was selected to facilitate a notable increase in the specific Faraday rotation, whilst at the same time avoiding a significant reduction in the upper limits of the annealing temperature range. X-ray diffraction (XRD, Bruker D5000, Siemens AG, Munich, Germany) measurements (performed in the range of 2θ angles between 20° and 70°, as shown in Figure 4b) revealed the body-centered cubic lattice structure of different constituent garnet materials present inside annealed trilayers. Their microstructure type was identified as being nanocrystalline on both substrate types. A notable and unexpected feature of hysteresis loop behavior (Figure 4c) was the presence of an “intermediate saturation” state exhibiting the maximum Faraday rotation, which then was followed by conventional magnetization saturation state with reduced Faraday rotation, at increasing external magnetic fields above about 1 kOe. The final saturated Faraday rotation was observed near 1.6 kOe, below 50% of the maximum Faraday rotation angle seen at smaller fields. To the best of our knowledge, no prior (or later) reports of similar magnetic switching behaviours observed in any thin-film materials, relative to [80], can be found in the published literature. These exchange-coupled all-garnet multilayer structures demonstrated an attractive combination of optical and magnetic properties and are of interest for emerging applications in optical sensors and isolators, ultrafast nanophotonics, magneto-plasmonics, and possibly spintronics.

The objective of making this all-garnet trilayer structure was to investigate the potential of obtaining and controlling these special magnetic switching properties observed in the described trilayer types that are not attainable normally in any single-layer magnetic thin films. We note that the unconventional magnetic behavior has only been observed in trilayers employing cosputtered nanocomposite-type outer magneto-hard layers. Further studies are ongoing, aimed at achieving better control over the magnetic properties (e.g., coercive force, switching field(s), and saturation magnetization) in garnet multilayer structures having different combinations of high-performance garnet materials of various optimized thicknesses as well as stoichiometry types. Other active areas of ongoing research within our group include the investigations of the effects of the postdeposition oxygen plasma treatment of amorphous-phase garnet precursors on the final magnetic and MO properties observed in crystallized films. Initial findings indicate that there exist optimized oxygen plasma posttreatment regimes, which lead to observing strong improvements in the MO quality of annealed thin garnet layers.

## 3. Ruthenium Oxide Thin-Films Grown on Flexible and Rigid Substrates for Cost-Effective Sensor Electrodes

Sputter deposition process parameters, such as total or partial gas pressure(s), process gas chemistry, substrate type, substrate-stage heating temperature, substrate-stage rotation rate, substrate-to-target distance, target rotation rate, and RF powder densities, are well known to affect the growth and properties of thin-film materials. There are many reports published in the literature describing the necessity of sputter deposition process parameter optimization for many application-specific oxide-based and other thin-film materials [81,82,83,84,85,86,87,88,89,90,91]. For example, sputter deposition process parameters were studied and optimized to deposit ruthenium oxide (RuO_2_) thin films with strong adhesion properties for manufacturing durable sensing electrodes [87,91]. For the manufacture of sensors, it is important to control the material properties in order to obtain the desired functionality [92]. In this section, we present optimized RF sputter deposition process parameters that were used to deposit highly durable RuO_2_ thin-film electrodes on flexible and rigid substrates, along with the pH and temperature sensing performance of these electrodes.

Ruthenium oxide thin films feature very attractive properties for use in various sensing devices, such as pH sensing, cryogenic temperature sensing, and even biosensing applications [93,94,95]. In order to fabricate durable and therefore cost-effective electrodes, it is important to have strong adhesion of RuO_2_ to its substrate material. This requires optimization of multiple sputter deposition process parameters to overcome sputter chamber pressure-dependent adhesion properties of RuO_2_ layers fabricated on different substrates [93].

### 3.1. Experimental

**Figure 5:**
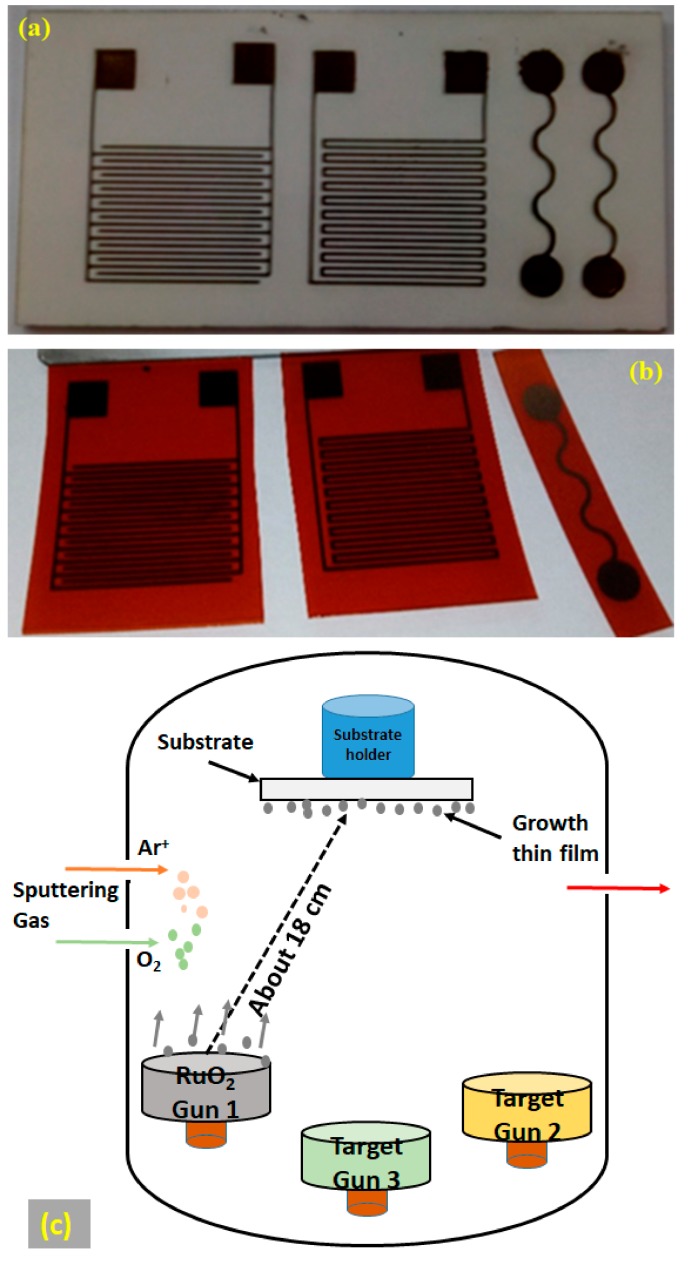
Potentiometric, interdigitated, and resistive RuO2 electrodes, 100 nm thick, sputter-deposited on (**a**) Alumina; (**b**) Polyimide substrates; and (**c**) schematic diagram of the up-sputtering system geometry.

RuO_2_ thin-film electrodes (potentiometric, interdigitated, and resistive) were deposited directly onto two different substrate materials (alumina and polyimide), as shown in Figure 5. In summary, 100 nm thick layers of RuO_2_ were deposited using RF magnetron sputtering from a metal oxide (RuO_2_) target (99.95% purity) with 110 W sputter power, at room temperature, and with 4 mTorr chamber pressure. From our previous experience with RuO_2_ thin film growth on different substrates, e.g., Al_2_O_3_ and glass, we found that the films possessed better crystallinity if they were prepared with total process-gas pressure at above 4 mTorr, with ≥50% of oxygen partial pressure in the plasma [93]. We adjusted the partial pressure ratio for Ar and O_2_ to be as close as possible to 1:1, by way of adjusting the partial gas flow rates, which were measured by the flow rate meter. Several batches of high-quality RuO_2_ films were deposited by using an RF magnetron sputtering system (KVS-2004L, Korea Vacuum Technology Ltd., Gyeonggi-do, Korea). At least a dozen process parameter optimization runs were performed, while depositing test film batches, adjusting the various experimental parameters (e.g., the partial gas pressures and RF power densities), and making process calibration experiments, before the final high-quality batches of RuO_2_ films were fabricated. The oxide-based target material (RuO_2_) of 4-inch (10.16 cm) diameter was placed at one of the guns of the up-sputtering system, having about 18 cm of source-to-substrate separation, as shown in the schematic diagram (Figure 5c). In this sputtering system, all the guns with independently activated shutters are placed at the corners of the equilateral triangle and tilted towards the substrates. The substrates’ stage rotation was adjusted (as a result of prior process optimization efforts) to be near 10–11 rpm during the deposition processes, and the film thicknesses were monitored during the deposition using a calibrated microbalance quartz sensor. Electrode patterns were achieved using a standard photolithographic process.

Potentiometric measurements were made using an Atlas Scientific LLC (Long Island City, NY, USA) Oxidation and Reduction Potential “ORP EZO” circuit connected to PC via an Electrically Isolated “USB EZO” Carrier Board, with a commercially available glass Ag|AgCl|3 M KCl reference electrode (Sigma-Aldrich, Sydney, Australia). Resistive electrodes were characterized with an Agilent 34410A high-performance digital multimeter, using a 2-wire setup to measure resistance. Measurements were made using commercially available buffer solutions (Rowe Scientific, Perth, Australia) for pH. Meanwhile, temperature was controlled using a thermoelectric device and insulation.

### 3.2. Results and Comparison

**Figure 6:**
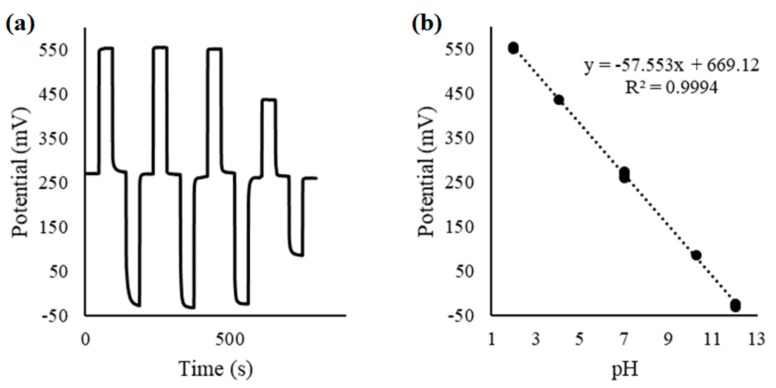
Electric potential recorded vs. a Ag|AgCl|KCl reference electrode for the potentiometric RuO_2_ electrode on polyimide substrate, in pH 2, 4, 7, 10, and 12 buffers (**a**); and the reversible linear Nernstian pH response of the electrode (**b**).

**Figure 7:**
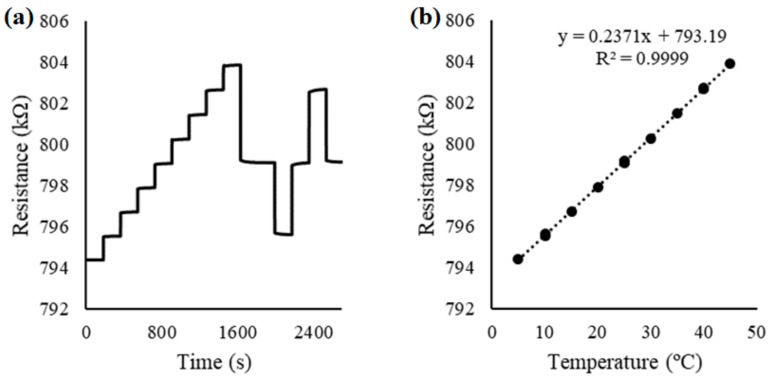
Electric resistance variations recorded for a RuO_2_ electrode on alumina substrate, at 5 to 45 °C (**a**); and the reversible linear temperature response of the electrode (**b**).

RuO_2_ films sputter-deposited directly onto flexible polyimide substrates can be a cost-effective choice for use as a potentiometric electrode. It was found experimentally that the polyimide-based electrodes prepared using a 4 mTorr chamber pressure and 1:1 Ar:O_2_ gas ratio showed excellent performance when used as potentiometric pH electrodes (vs. Ag|AgCl|KCl reference electrode), exhibiting a reversible (±3.9 mV), linear (R^2^ = 0.9994), and Nernstian (57.5 mV/pH) pH response, as shown in Figure 6. The obtained result is comparable to previously reported RuO_2_ pH electrodes prepared on alumina substrates, which also exhibited a reversible (±1.8 mV), linear (R^2^ = 0.9999), and Nernstian (59.1 mV/pH) pH response [93]. The polyimide-resistive and interdigitated electrodes failed when used as conductivity and temperature sensors, due to gradual delamination. However, the alumina-based resistive temperature sensor exhibited a reversible (±0.01 °C), linear (R^2^ = 0.9999) response, which is comparable to some commercially available sensors as shown in Figure 7.

To summarize, durable metallic RuO_2_ thin films could only be deposited with a chamber pressure ≥4 mTorr with argon–oxygen content in the chamber. RuO_2_ films deposited at pressures lower than 4 mTorr produced powdery films that rapidly delaminated from all substrates. The improved durability of these RuO_2_ electrodes allows for the development of reliable sensors, potentially for miniaturised applications or where conventional glass probes are not suitable, such as biomedical applications.

Other RF-sputtered metal oxide materials investigated by our group included rare-earth oxide compositions such as Er_2_O_3_, Dy_2_O_3_, Gd_2_O_3_, and others, e.g., Zr_2_O_3_, TiO_2_, SnO_2_, and Al_2_O_3_. Intended applications included the scratch-proof and hydrophobic film layers, sensing applications, diffusion protection layers, and generic multilayer constituents. Two principal categories of deposition processes were explored with oxide materials: Sputtering in pure-argon plasma and the processes run using argon–oxygen plasma mixes. Substantial differences in the resulting oxide material properties were observed, depending on the deposition process type used. The main reason for the observed property differences was the oxygen loss occurring during sputtering deposition, which has often been possible to recover using high-temperature oven annealing processes.

## 4. Metal–Dielectric Nanocomposite-Based Multilayer Building Blocks for Transparent Solar and Thermal Regulation Coatings

The development of modern energy-efficient glass and window products requires specialized thin-film coatings containing silver (Ag) layer(s) to filter or reflect the infrared and far-infrared (thermal) radiation components of the solar spectrum, thus reducing the energy consumption in buildings. Furthermore, surface plasmon resonance-based optical sensors require noble metal-dielectric interfaces and have numerous commercial applications in gas, biochemical, and chemical sensing due to their high sensitivity to the refractive index changes in a dielectric medium placed on top of ultrathin metallic (Ag) films [96,97,98,99]. The deposition of the ultrathin Ag layers with low surface roughness and high uniformity is the key to achieving the required application-specific optical properties. However, the deposition of ultrathin (less than 20 nm) Ag layers often results in forming morphologically uneven nanoislands, even when using the fine-tuned deposition processes, such as RF magnetron sputtering [100,101]. The formation of these Ag nanoislands significantly affects the optical performance of multilayer metal–dielectric thin-film structures. Typically, reduced reflectivity across the near-infrared range results from the imperfect metal layer morphologies. In addition, dielectric material layers deposited on top of these island-shaped ultrathin Ag layers tend to “sink” into the spaces between these silver nanoislands, leading to strong optical property deviations from the designed response. Another issue associated with Ag is that it oxidizes very quickly; therefore, the Ag layer has to be protected from oxidation, even during the deposition of the next material layer in the thin-film multilayer sequence. Researchers have proposed and trialled the preparation of nanocomposites to overcome these issues related to the difficulties of forming ultrathin Ag layers. Most of the relevant success stories were about depositing metal–dielectric nanoparticle-based composites [102,103,104,105,106].

We prepared cosputtered metal-dielectric nanocomposites by developing a cosputtering process for an Ag-MgF_2_ system, with various volumetric contents of the dielectric phase added to the metal. We also developed MDC-containing multilayer structures, and their characterisation results suggest that MDC layers can replace the pure-metal layers in multilayer optical coatings, thus offering a new possibility to design and fabricate highly durable and environmentally stable coatings on glass, suitable for solar and thermal regulation technologies [107]. Of particular importance are the forward-looking emergent and future applications of MDC-based highly stable, high-transparency, low-emissivity (heat-mirror-type) coatings in novel areas, such as solar window development [108]. High-transparency, energy-generating, and simultaneously energy-saving solar window products are currently at the cutting edge of the worldwide product development efforts in building integrated photovoltaics (BIPV), which itself is an area of rapid growth in both research and the marketplace [109]. In this section, we present the growth process details for thin-film single-layer MDC and newly developed MDC-containing multilayer coatings. Their measured optical characteristics are also reported. The transmission spectrum features of these newly developed MDC-based multilayer building blocks appear to be promising for manufacturing cost-effective solar and thermal heat regulation coatings for the construction industry in the near future.

### 4.1. Deposition of MDC Layers and Multilayer Structures

**Figure 8:**
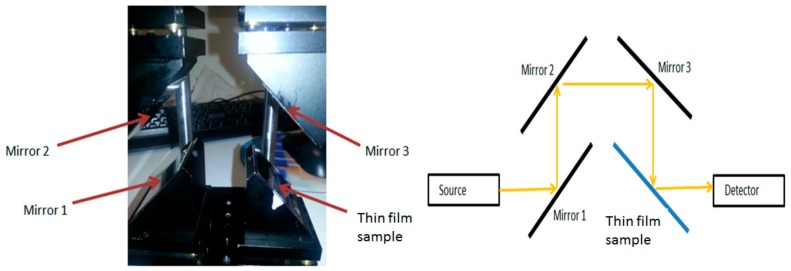
Schematic diagram of the optical system used in Cary 5000 spectrophotometer to measure the reflection spectrum of the thin-film coatings on glass substrates.

Several batches of MDC (Ag/MgF_2_) films were deposited onto glass substrates by using the cosputtering process in an RF magnetron sputtering system. The volumetric fraction of dielectric (MgF_2_) phase was controlled by adjusting the RF power densities applied to the MgF_2_ sputtering target during the cosputtering process, after carefully calibrating the partial deposition rates for both MgF_2_ and Ag. The thicknesses of ultrathin composite films trialled were in the range between 12 and 25 nm. After the successful growth of MDC layers, we prepared several multilayer structures, introducing the optimized MDC layers along with surrounding oxide layers. Table 2 summarizes the deposition process parameters used to fabricate these metal-dielectric nanocomposite layers and MDC-containing multilayer building blocks. The transmission and reflection spectra of MDC layers were measured just after the deposition using an Agilent Cary 5000 UV-VIS-NIR spectrophotometer (Agilent Technologies Australia Pty Ltd., Sydney, Australia). The reflection spectrum measurements were carried out using a custom-built reflection-mode optical measurement module mounted inside the spectrophotometer, which contained four mirrors adjusted at 45° tilts to form a periscope-type device as shown in Figure 8.

Since our primary motivation for MDC materials development was to broaden the range of possible high-performance designs of heat-mirror-type coatings, and because the reflectivity of these coating types across the near-infrared range is their main performance indicator, developing both the transmission and reflection spectral measurements was necessary.

### 4.2. Optical Characteristics of MDC Thin Films and MDC-Containing Multilayer Structures

**Figure 9:**
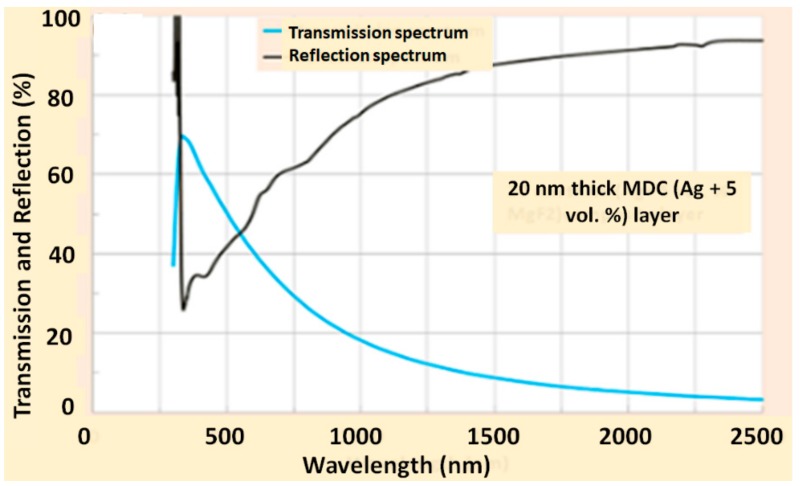
Measured transmission and reflection spectra of ~20 nm thick Ag + 5 vol. % MgF_2_ composite layer, deposited onto glass substrate.

Figure 9 shows an example of the measured transmission and reflection spectrum of Ag/MgF_2_ (5 vol. %) composite (~20 nm) thin film. The transmission peak in this Ag/MgF_2_ composite layer exhibited short-wavelength transmission and reflection features similar to the Ag layer of the same thickness. The optical absorption was calculated by subtracting the transmittance and reflectance of the composite layers using the formula A = 1 − T − R (%), where A is the absorbed power fraction, T is the power transmission coefficient, and R is the power reflectivity. It was found that the Ag + 5 vol. % MgF_2_ sample had a comparatively lower absorption (less than 9 % by power) in the UV-Visible spectral range, which is close to that of the pure Ag layer. This indicates that the addition of 5 vol. % MgF_2_ into the Ag layer during the cosputtering process can potentially improve the coating adhesion and stability aspects without compromising the plasmonics metal properties.

**Figure 10:**
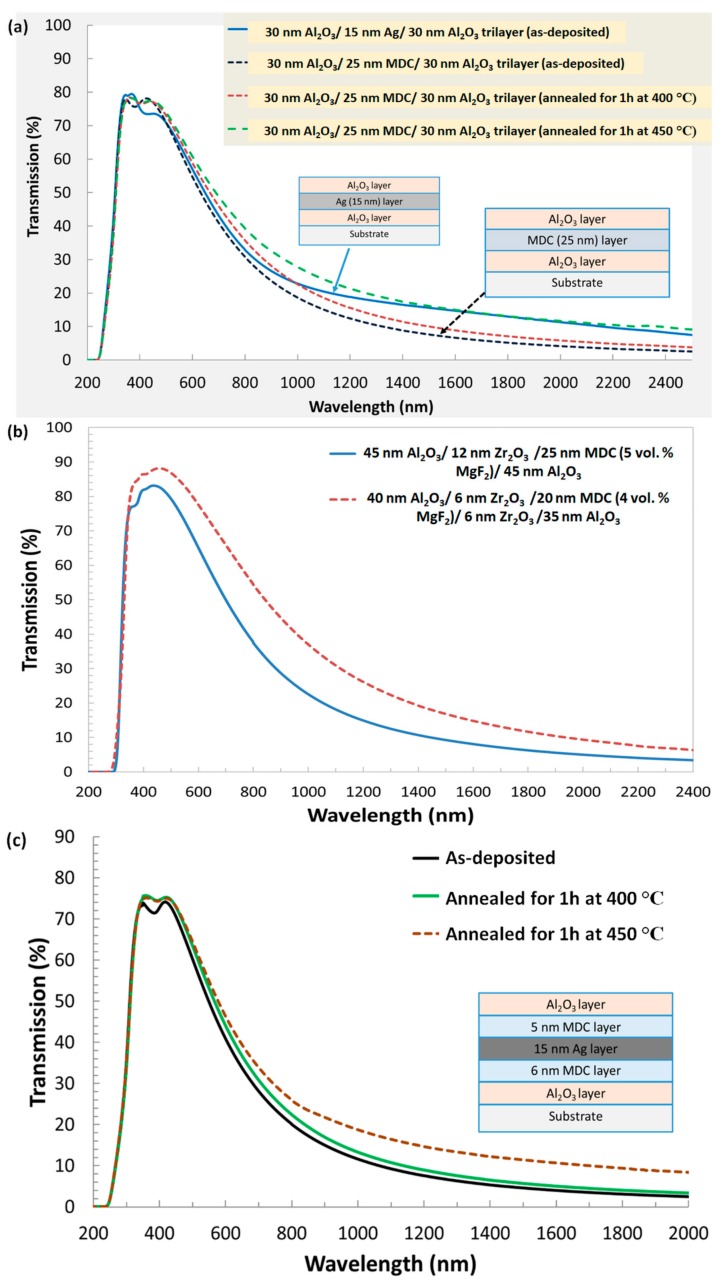
Measured transmission spectra of multilayer thin-film structures containing a single (or double) metallic or MDC layer. (**a**) Transmission of a trilayer MDC structure compared to that of a typical Ag layer within a dielectric–metal–dielectric (DMD) structure; thermal performance stability test results are also shown for the trilayer MDC structure; (**b**) transmission spectra of multilayer structures containing MDC layer of different volumetric fractions of added dielectric; and (**c**) thermal performance stability test results for a complex DMD-type multilayer structure containing both the MDC outer-bracket sublayers and an ultrathin pure-Ag core layer.

Several trilayer structures containing at least one MDC layer were deposited on clear glass substrates using one continuous sputtering run, and their optical performance was characterized. Figure 10a shows the transmission spectrum of as-deposited multilayer coatings, where the layer structure consists of at least one MDC (Ag + 4–5 vol. % MgF_2_) in comparison to that of a typical trilayer pure metal (Ag)-based DMD-type coating. It can be noted that the use of a 25 nm MDC layer instead of a pure 15 nm Ag layer resulted in better thermal radiation filtering performance without compromising the visible spectral transmission, whilst avoiding any potential layer morphology-related issues that usually affect ultrathin (thinner than about 17 nm) layers. Experimental investigations revealed that the cosputtered MDC (even comparatively thicker) layers with high metal volume content could be embedded into any type of multilayer structures for possibly improving the spectrally selective transparency control and structural properties of the coating, whilst allowing significant design flexibility. Particularly important is the possibility of depositing MDC-type layers onto practically any underlying film layers, almost regardless of surface energy-related layer interface compatibility considerations. The MDC-based structures also demonstrated much lower thermal emissivity, whilst maintaining the same superior visible transmission compared to the silver-based coatings, as shown in Figure 10a.

In order to investigate the design variations, material intercompatibility, durability, and thermal stability properties of the MDC-based coatings, several different types of test multilayers (up to five-layer structures) were developed, as shown in Figure 10b,c. Figure 10b shows the optical performance of an elementary (five layer-based) thin-film coating building blocks composed of a center MDC layer, surrounded by thin oxide-based “bracket layers” deposited by a pure-argon sputtering process from a ceramic oxide target of Zr_2_O_3_. The addition of these ultrathin rare-earth oxide layers broadens the structure design possibilities in terms of improving the peak transmission and spectral selectivity. The optical performance variations observed in these particular five layer-based coatings confirmed the possibility of engineering a broader range of optical coatings for future solar and thermal regulation application in comparison with simple dielectric-metal-dielectric (DMD) trilayers or multilayers of similar spectral response. Figure 10c presents a more complex, generalized, MDC-type multilayer building block, which was subjected to extensive thermal stability (dry heat exposure) tests. The performance of the developed structure (before and after heat exposure in an air-filled lab oven) shows that the optical properties are comparable to some of the conventional multilayer structures containing pure Ag layers [110,111]. It can be seen clearly that the transmission spectra were found to be only slightly changed at temperatures near 400 °C, which is a very encouraging result, and confirms that the very thin MDC barrier layers can play a vital role in protecting the ultrathin core Ag layers within the multilayer structures. This confirms that the MDC layers can be used to enable arbitrarily complex high-stability low-E, heat-mirror or filter-type optical coating designs of high spectral selectivity, on a range of optical substrates. The low thermal emissivity property of heat-reflecting multilayers appears as a consequence of their high reflectivity across the broad spectral range from the near-infrared to the far-infrared wavelengths (in excess of several tens of μm). The energy-saving properties in these low-E coatings arise from keeping the thermally emitted wavelengths reflected inside building spaces, thus improving the thermal insulation properties in purpose-designed glazing systems. Additionally, it is possible to engineer a broader range of reflected or transmitted colour properties, compared to pure metal-based designs, which are much more limited in terms of the optical materials selection.

## 5. Conclusions

We demonstrated a range of material system design possibilities for multiple practical applications provided by fine-tuning the RF magnetron sputtering technologies. We established numerous sputtering-based thin-film material synthesis processes at ECU, aiming at incorporating these optical materials into functional devices for sensing, optical spectra shaping, polarisation control, magnetic switching, and magnetic field visualization, among others. Several previously unforeseen and underexplored properties and behaviours were identified in functional materials and structures deposited by sputtering. Among these is the unconventional hysteresis loop behaviour first observed in nanocomposite-based magneto-optic garnet multilayers, where an intermediate saturation-type magnetization state between ~700 and 1100 Oe existed. Additionally, we observed a rather high (up to >400 °C) thermal exposure (dry heat) stability in nanoengineered metal–dielectric multilayers. A cost-effective approach to the development of ruthenium oxide-based pH sensors on flexible substrates was also reported. Three functionally different categories of thin-film material systems were discussed, such as MO garnets, metal oxides, and metal–dielectric nanocomposites, and results were presented describing their growth processes and relevant parameters, physical properties, and behavior. All of the characteristic results obtained from these material system types are of interest for use in practical field-specific applications in optics, magneto-optics, sensing, and renewable energy.

## Figures and Tables

**Table 1:** Sputtering process parameters used to deposit garnet, garnet-oxide precursors (as single layer films), and all-garnet multilayer structures.

Process Parameters	Layer Structure (Bi-Substituted Iron Garnet and Garnet-Oxide (Single) Layer)	Layer Structure (GOC-Based All-Garnet Multilayers)
Sputtering targets	Bi_2_Dy_1_Fe_4_Ga_1_O_12_ and Bi_2_O_3_	Bi_2_Dy_1_Fe_4_Ga_1_O_12,_ Bi_1.8_Lu_1.2_Fe_3.6_Al_1.4_O_12_ and Bi_2_O_3_
Radio frequency (RF) power	Bi_2_Dy_1_Fe_4_Ga_1_O_12_ (150–300 W), and Bi_2_O_3_ (40–150 W)	Bi_2_Dy_1_Fe_4_Ga_1_O_12_ (300 W), Bi_2_O_3_ (60 W), and Bi_1.8_Lu_1.2_Fe_3.6_Al_1.4_O_12_ (170 W)
Base pressure (Torr)	<3 × 10^−6^–5 × 10^−6^	<3 × 10^−6^–5 × 10^−6^
Process gas	Ar	Ar
Process pressure	2–3 mTorr	2–3 mTorr
Target-to-substrate distance (cm)	~18	~18
Substrate stage rotation rate (rpm)	15–16	15–16
Substrate temperature (°C)	Room temperature	Room temperature

**Table 2:** Sputtering process parameters used to deposit metal–dielectric nanocomposite (MDC) single layer and MDC-containing multilayer building blocks.

Process Parameters	Layer Structure (Single Layer MDC)	Layer Structure (Multilayers Containing MDC Layer)
Sputtering targets	Ag, MgF_2_	Al_2_O_3_, Zr_2_O_3_, Ag, MgF_2_
RF Power	Ag (55–65 W), MgF_2_ (83–95 W)	Al_2_O_3_ (190–195 W), Ag (55–65 W), MgF_2_ (83–95 W)
Base pressure (Torr)	5 × 10^−6^	5 × 10^−6^
Process gas	Ar	Ar
Process pressure	2–3 mTorr	1–3 mTorr
Substrate temperature (°C)	Room temperature	Room temperature
Post deposition heat treatment	No	Up to 500 °C for 1–10 h
